# Demographic analyses of a new sample of haploid genomes from a Swedish population of *Drosophila melanogaster*

**DOI:** 10.1038/s41598-020-79720-1

**Published:** 2020-12-29

**Authors:** Adamandia Kapopoulou, Martin Kapun, Bjorn Pieper, Pavlos Pavlidis, Ricardo Wilches, Pablo Duchen, Wolfgang Stephan, Stefan Laurent

**Affiliations:** 1 Institute of Ecology and Evolution, University of Bern, Baltzerstrasse 6, 3012 Bern, Switzerland; 2 Department of Evolutionary Biology and Environmental Sciences, University of Zurich, 8057 Zurich, Switzerland; 3 Division of Cell and Developmental Biology, Medical University of Vienna, 1090 Vienna, Austria; 4 Département de Biologie, Université de Fribourg, 1700 Fribourg, Switzerland; 5 Department of Comparative Development and Genetics, Max Planck Institute for Plant Breeding Research, 50829 Köln, Germany; 6 Institute of Computer Science, Foundation for Research and Technology-Hellas, Crete, Greece; 7 Section of Evolutionary Biology, Department of Biology II, University of Munich, 82152 Planegg, Germany; 8 Department of Computational Biology, Université de Lausanne, Lausanne, Switzerland; 9 Leibniz Institute for Evolution and Biodiversity Science, Natural History Museum, 10115 Berlin, Germany

**Keywords:** Population genetics, Molecular evolution

## Abstract

European and African natural populations of *Drosophila melanogaster* have been the focus of several studies aiming at inferring demographic and adaptive processes based on genetic variation data. However, in these analyses little attention has been given to gene flow between African and European samples. Here we present a dataset consisting of 14 fully sequenced haploid genomes sampled from a natural population from the northern species range (Umeå, Sweden). We co-analyzed this new data with an African population to compare the likelihood of several competing demographic scenarios for European and African populations and show that gene flow improves the fit of demographic models to data.

## Introduction

*Drosophila melanogaster* originated in sub-Saharan Africa where it diverged from its sister species *Drosophila simulans* approximately 2.3 million years ago^1^. The species has a strong dispersal capacity and long-distance migration has been reported in field experiments^2^. Previous genetic analyses of European and Asian samples indicated that non-African populations started expanding beyond their ancestral range around 13,000 years ago, eventually colonizing large areas in Europe and Asia^3–6^. By contrast, the introduction of the species in the Americas and Australia is very recent (within a couple of hundred years ago) and has been well documented by early entomologists (Keller^7^). Interestingly, demographic analyses of North-American and Australian populations revealed significant African ancestry (between 15 and 40%) in a dominantly European background^8–11^. However, less attention has been given to gene flow between African and European samples (but see Medina et al.^12^, Arguello et al.^13^).

Recent advances in sequencing technology have led to a dramatic increase of genome-wide studies of genetic variation in natural populations. Particularly in *Drosophila melanogaster*, international research groups and consortia are generating large-scale genomic datasets from natural populations, which are densely sampled through time and space^14^. The major aim of these studies is to understand the evolutionary history of broadly distributed populations and to identify signals of adaptation in response to spatially or temporarily varying environments^15–20^. Analyses of comprehensive genomic datasets have, for example, only recently revealed previously unknown population structure along the East–West axis in Europe^17,21^ or the predominant effect of inversions on clinal variation along the East coasts in North American^18,22^ and Australia^23^. However, a major hurdle of such population genetic approaches is the urgent need for accurate null models which require a fundamental understanding of the demographic history^24^. We believe that our analyses, which provide new and more accurate estimates of key demographic parameters, will prove useful in future genome-wide studies to investigate the evolutionary history of non-African *Drosophila* populations and to distinguish signals of adaptation from neutral evolution.

Here, we followed a protocol proposed by Langley et al.^25^ and sequenced haploid genomes of 14 *Drosophila melanogaster* embryos obtained from a Swedish population. We describe patterns of genetic diversity and compare them to previously available data from a Zambian population located in the ancestral range of the species. We use this new dataset to test for the importance of migration in the demographic history of European populations and show that accounting for historical gene flow in demographic models of European and African populations significantly improves the fit to the data compared to models without gene flow.

## Results

**Figure 1:**
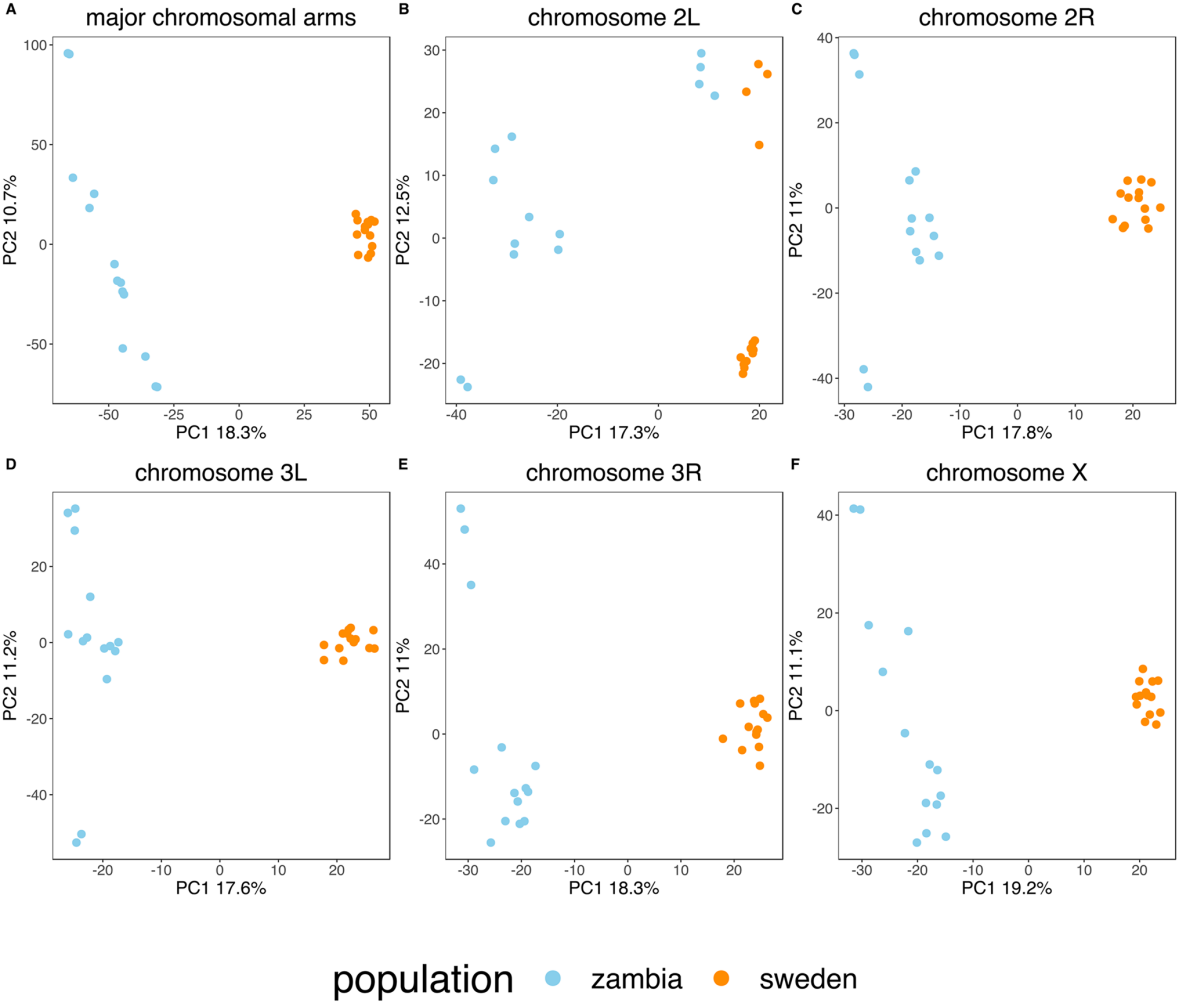
PCA results. Results are presented for each major chromosomal arm separately and for all chromosomes together. Only the first two components are shown. Individuals tend to cluster according to their sampling location (**A**, **C**–**F**) except for chromosome *2L* (**B**), for which the inversion *In(2L)t* creates an additional level of clustering.

Previous studies showed that ancestral lineages of current European flies diverged from sub-Saharan populations at the end of the last glacial maximum^26^ and that the colonization was associated with a population size bottleneck^4,27^. We conducted a principal component analysis (PCA) to explore whether our new genome-wide data is consistent with those expectations (Fig. 1). Our results based on the full dataset showed that the first principal component (PC1) consistently separated the European and African samples and that Swedish lines were consistently less dispersed, reflecting lower diversity compared to the Zambian sample (Table S1). One important exception to this pattern was observed on chromosome *2L*. In addition to the continent-specific separation on PC1, we identified an equally strong clustering on PC2, caused by the common cosmopolitan chromosomal inversion *In(2L)t*, for which high genetic differentiation has already been reported between standard and inverted haplotypes^28^*.* Therefore, SNPs from chromosomal arm *2L* were excluded from further demographic analyses. Summary statistics of polymorphism data were in line with previously published values for European samples, with lower nucleotide diversity in Sweden (θ_w_ = 0.003) than Zambia (θ_w_ = 0.006, Table S1,^29^).

The demographic inference methodology implemented in *dadi*^30^ assumes that the genetic variation used for model optimization is not affected by negative selection, which can create biases in demographic analyses^31^. Because the magnitude of indirect negative selection is known to depend on the local recombination rate^32^ we partitioned our data into three classes of recombination ([0,1.5], [1.5,3], [3,14.5] cM/Mb) based on published estimates^33^ and compared the site frequency spectrum (SFS, the distribution of allele frequencies) between recombination classes to identify signatures of linked negative selection. In the low-recombination class, we identified a significant skew in the SFS of the autosomal data towards low-frequency classes (Pearson’s chi-squared test: p < 0.001, Fig. S1), which is consistent with the predicted effect of negative selection on linked neutral variants^34^. Consequently, we restricted our analyses to regions with local recombination rates larger than 1.5 cM/Mb.

**Figure 2:**
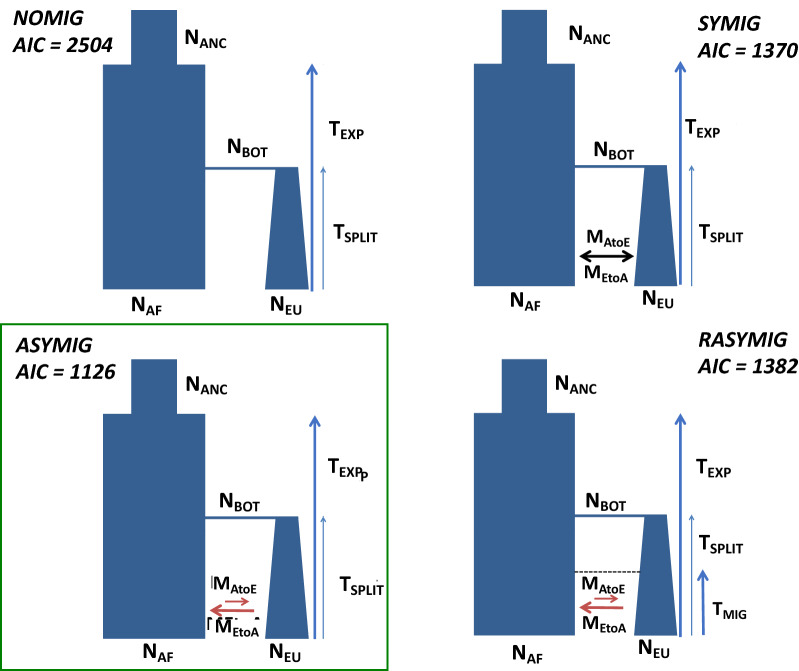
Results of the model choice analyses with the four demographic models tested in this study. Lowest AIC out of 10 replicates are reported for each model (autosomal data only). The green box with a continuous line indicates the best model for the autosomal and X-chromosomal data. Abbreviations in the figures are explained in the Materials and Methods section and Table 1.

Demographic inference conducted with *dadi* showed that models with migration provided a better fit than models without migration, for both the autosomal and X-linked data (Fig. 2, Figure S2-4). This method allows to conduct model-based inference based on the SFS and can therefore be used to identify models and numerical values for demographic parameters that generate the best possible fit between theoretical and empirical SFS. For this analysis we compared four possible demographic scenarios. Model 1 (NOMIG) does not implement gene flow and is therefore similar to previously published models^3,4,9^. Model 2 (SYMIG) implements symmetrical migration between the populations, starting immediately after the split and lasting until the end of the simulation (present). Model 3 (ASYMIG) is like model 2 but allows for asymmetrical migration rates. Finally, Model 4 (RASYMIG) is similar to model 3 except that asymmetrical migration only starts at time T_mig_. For autosomal and X-linked data, the best model was ASYMIG in which asymmetrical migration is ongoing since the divergence time. The identification of gene flow as a relevant parameter in demographic models of *Drosophila melanogaster* has been reported before^8,13,35,36^ and leads to older estimates of population split times, compared to estimates obtained using models without migration (Table S2).

Conversion of maximum-likelihood estimates to population sizes and years requires independent information about mutation rate and generation time. Our previous studies relied on divergence-based estimates of the mutation rate and the assumption of 10 generations per year^3,9,13^. However, empirical measurements of the mutation rate using mutation accumulation lines yielded a mutation rate of 5.21 × 10^–9^ for single nucleotide substitutions on autosomes^37^, representing a 3.75-fold increase compared to the estimate used in Arguello et al.^13^. Furthermore, Pool (2015) estimated a generation time for natural populations of *D. melanogaster* of 15 generations per year, which is supported by larger empirical evidence than the previously used estimate of 10 generations per year. Demographic estimates based on autosomal data for our best model were in line with previously published estimates when using the same estimates for mutation rate and generation time (Table S2,^13^). Naturally, the use of a larger mutation rate and a shorter generation time yields smaller population sizes and younger ages for the split between the ancestral lineages of the European and African samples (T_split_ = 4,139 years) as well as the African expansion (13,032 years, Table 1). Similar results have been recently published by Sprengelmeyer, et al.^38^ and altogether suggest that the spread of populations outside their center of origin may have coincided with the Neolithic demographic transition. Estimates using X-chromosomal data are markedly different from autosomal data, which likely reflects selective processes specifically affecting sex-chromosomes^39^.

**Table 1:** Demographic estimates for the best model (ASYMIG).

Parameters	Autosomes			X		
	Max-likelihood estimate	CI-lower bound	CI-upper bound	Max-likelihood estimate	CI-lower bound	CI-upper bound
Current African population size (N_AF_)	724,038	490,098	741,918	750,104	706,793	808,487
Current European population size (N_EU_)	155,202	67,444	633,186	59,031	35,093	106,347
European Bottleneck population size (N_BOT_)	15,817	3,252	45,664	2,935	1,905	4,341
Divergence of ancestral lineages (T_SPLIT_) years ago	4,139	2,466	13,039	1,429	1,144	1,715
African expansion time (T_EXP_) years ago	13,032	4,200	14,750	6,389	5,755	6,943
Ancestral African population size (N_ANC_)	177,344	172,652	202,222	110,240	108,307	112,659
Migration Rate Europe to Africa (MEtoA)	1.23	0.01	2.03	0.78	0.63	1.17
Migration Rate Africa to Europe (MAtoE)	0.4	0.05	1.03	0.9	0.72	1.11

Demographic estimates obtained with *dadi* for our best model (ASYMIG) for autosomal (*2R*, *3L*, *3R*) and X-linked. Estimates have been converted into years and population sizes assuming 15 generations per year and the mutation rate estimates of Huang et al. (2016). Migration rates are given in units of 2.N_ANC._m_ij,_ where *i* and *j* are the source and sink populations, respectively.

**Figure 3:**
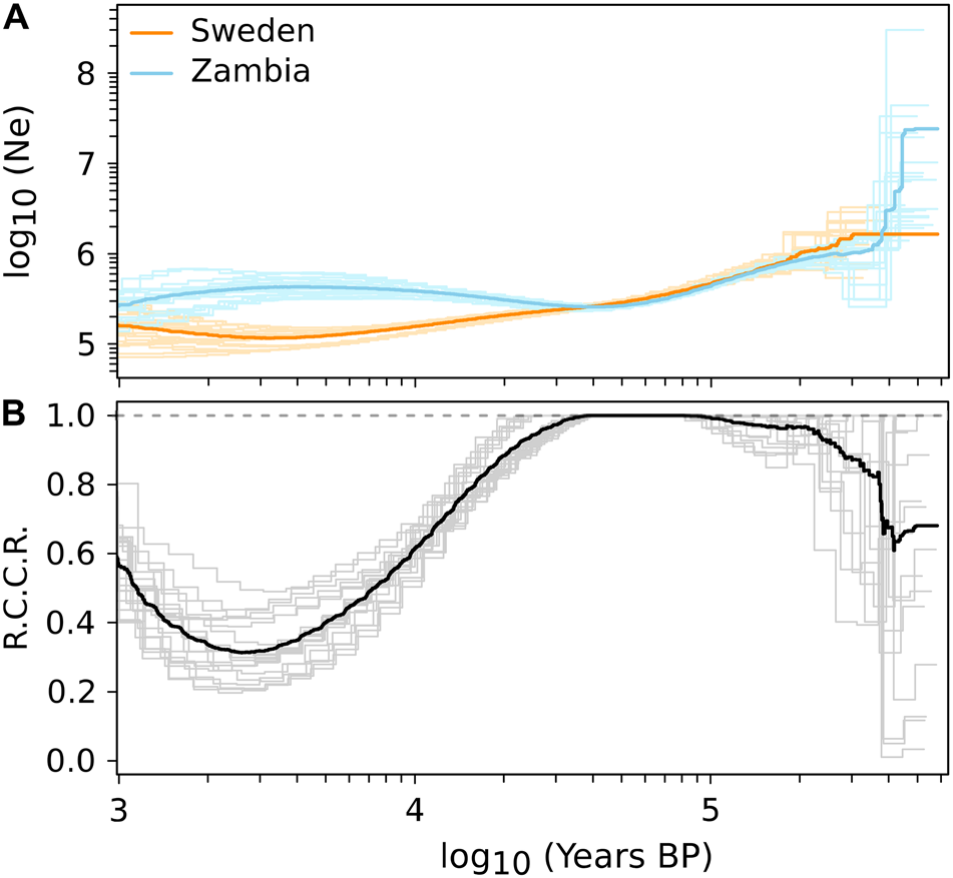
(**A**) Population sizes estimated by *MSMC2* for the Swedish (blue) and Zambian (orange) samples. Population sizes were obtained by rescaling coalescence rates using the empirical mutation rate of Huang et al. (2016), 5.21 × 10^–9^. The x-axis represents time in years assuming a generation time of 15 generations per year (Pool 2015). In the more distant past, both curves display identical sizes suggesting a common ancestral population. (**B**) RCCR: Relative cross-coalescence rates between the Swedish and Zambian samples. RCCR = 0 indicates full-isolation between both populations and RCCR = 1 indicates a panmictic population.

As a complementary approach to our SFS-based demographic estimations, we also estimated coalescence rates for the autosomal data within and between populations using the software *MSMC2*^40,41^. This method can be used to estimate continuous changes in population size through time by applying a Markovian approximation of the coalescent-with-recombination process. This method can, unlike *dadi*, account for the dependencies between linked SNPs and therefore obtain demographic information from both coalescence and recombination processes. Estimated coalescence rates were rescaled into population sizes using the mutation rate of Huang, et al.^37^ (Fig. 3A). While population sizes and the ages of events largely agreed between both methods, the *MSMC2* results did not identify the presence of a population size bottleneck in the European population and also indicated a recent increase in gene flow between the European and African samples (Fig. 3B), which is not predicted by our best demographic model in the *dadi* analyses (Fig. 2).

## Discussion

Our PCA for chromosome *2L* illustrates the influence that inversions can have on polymorphism data. Importantly, the structure created by *In(2L)t* extends several megabases beyond the inversion’s breakpoint^28,42^. Therefore, we excluded all of chromosome *2L* for the demographic analyses in this study and recommend that future demographic studies of natural populations of *Drosophila melanogaster* address the potential effect of this inversion prior to demographic inference. We also identified an excess of singletons among low-recombining regions (smaller than 1.5 cM/Mb) in the autosomal data of our Swedish sample, which is likely caused by linked negative selection and should therefore be filtered out when conducting demographic inference.

Early studies about the demographic history of European populations did not consider the effect of gene flow when estimating the age of the population split^3–5^. However, Li and Stephan^4^ already predicted that accounting for gene flow would lead to older divergence times, owing to the homogenizing effect of migration on allele frequencies in isolated populations. Our results confirm this prediction (Table 1, Table S2) and show that taking gene flow into account almost doubles the estimated divergence time (2247 for model NOMIG vs. 4139 for model ASYMIG, Table S2). However, estimations presented in this study (Table 1) are substantially lower than previous estimations of the divergence time (despite taking gene flow into account) because different mutation rates and generation times were used to rescale the timing of demographic events from coalescence units into years. While increasingly sophisticated methods are improving the performance of demographic estimations, it is also becoming evident that empirical measurements of mutation rates or generation times are critical to assess the absolute age of evolutionary events. However, demographic models are often employed to predict neutral distributions of statistics of genetic variation that are then used as null-distributions in statistical tests of selection; and for such purposes, no information about mutation rates or generation times is required.

A major difference between the demographic histories estimated using *MSMC2* and *dadi* is the absence, in the former, of an obvious bottleneck in the ancestral lineages of the European sample. The existence of a population size bottleneck in the demographic past of cosmopolitan populations has been reported for the first time by Li and Stephan^4^ as well as Thornton and Andolfatto^27^ and has since been considered a major confounding effect for the detection of selective sweeps^24^. More work is needed in order to evaluate whether such bottlenecks are artefacts caused by over-simplistic parameterizations of demographic models or whether *MSMC2* (or similar methods) cannot detect such population size bottlenecks under specific conditions. This could be investigated by testing whether *MSMC2* can identify a bottleneck when used to analyze simulated datasets obtained from the ASYMIG model. Another promising avenue is *RELATE*, a recently published demographic inference method based on ancestral recombination graph (ARG) reconstruction^43^. Similarly to *MSMC2*, *RELATE* can be used to estimate continuous changes in population sizes but it also allows for the analyses of significantly larger sample sizes, which is expected to improve the quality of demographic inferences. It is also noteworthy that *MSMC2* appears to confirm the existence of a recent admixture event between African and European lineages while the RASYMIG model in the *dadi* analysis (in which gene flow could start after population divergence) provided a poorer fit to the observed data than the model with ongoing gene flow. More work is needed to identify whether this corroborates the earlier description of a recent event of cosmopolitan pulse-admixture into the African gene pool^12^ or rather reflects a loss of statistical signal in the most recent past. This work could be achieved by evaluating the performance of *dadi* and *MSMC2* using simulations with recent gene flow and by applying the recently published ARG-based method, which facilitate the estimation of gene flow and local admixture mapping^43,44^. *MSMC2* and *dadi* rely on different summarization of population genomic variation, which in principle could be capturing different aspects of the evolutionary signal; but it remains unclear how precisely this may contribute to the differences observed in this study.

## Materials and methods

### Data collection

A total of 96 inseminated female *D. melanogaster* were sampled from Umeå in northern Sweden in August 2012. Then, full-sibling mating was performed for 10 generations, which yielded 80 inbred lines. Out of these, 14 lines were randomly selected from which haploid embryos were generated following the protocol described by^25^. Standard genomic libraries were constructed using up to 10 μg (~ 200 ng/μl) of DNA. Library construction and sequencing of one haploid embryo for each of the 14 haploid-embryo lines were carried out on an Illumina HiSeq 2000 sequencer at GATC Biotech (Konstanz, Germany). In addition to the newly established and sequenced inbred lines from Umeå/Sweden, we randomly chose 10 lines not carrying any inversions from the DPGP3 dataset. They were collected in Siavonga/Zambia in July 2010 and sequenced as haploid embryos similar to our data. Since four of the Swedish lines carried the chromosomal inversion *In(2L)t*, we additionally chose four lines at random from Zambian lines that also carried *In(2L)t* to match the number and distribution of inversion karyotypes in our Swedish dataset. Accession numbers are listed in table S3. A detailed description of the bioinformatics methods used for mapping and variant calling can be found in the online supplementary text (Sup_text_NGS_methods.docx).

### Principal component analysis

Principal component analyses were conducted with the “auto_SVD” function from the R package *bigsnpr*^45^. This algorithm uses clumping instead of pruning to thin SNPs based on linkage disequilibrium, removes SNPs in long-range LD regions, and uses the thinned data to perform dimensionality reduction by singular value decomposition. Analyses were conducted on the full data and on each chromosomal arm separately.

### Demographic analyses

We used SNPs from introns with a local recombination rate larger or equal to 1.5 cM/Mb; autosomal and X-linked data were treated separately. Genomic regions spanned by common inversions were excluded from the analyses (as defined by coordinates of inversion breakpoints obtained from Corbett-Detig and Hartl^28^). Additionally, long runs of Identity-By-Descent were masked from the African lines using a perl script available from the DPGP website (http://www.johnpool.net/genomes.html). Genomic regions identified as of European ancestry in the DPGP2 and DPGP3 datasets were not masked, because our demographic analyses intended to evaluate the possibility of gene flow between the two populations. All coordinates were transformed to *D. melanogaster* genome reference version 6 using an in-house python script. In total, 4,020,733 bp (361,993 SNPs) were used for the three autosomes together and 5,859,268 bp (332,305 SNPs) for the chromosome X. We used the software *dadi*^30^ to test four different demographic scenarios. In all models, the ancestral African population experienced a stepwise expansion at time T_exp_. After the expansion, (forward in time) the European population splits from the African population at time T_split_. After the split, the size of the new European population is instantaneously reduced to a population size N_bot_, whereas the size of the African population does not change. After the bottleneck, the European population can recover exponentially until it reaches its current size N_eu_. The four scenarios differ in the modeling of migration following the population split. For every scenario, at least 10 independent runs with different initial parameter values were performed and the run achieving the highest likelihood was kept for parameter estimation and model choice. Model choice was done by comparing the Akaike information criteria (AIC) between models^46^. Confidence Intervals (CI) were calculated using the following procedure: First, 150 datasets were simulated using the best demographic model. These simulations were treated as pseudo-observed data and used to re-estimate demographic parameters under the best model. The set of 150 estimates for each demographic parameter was then used to construct the confidence intervals. Confidence intervals were calculated as the 2.5–97.5% percentiles. Nucleotide diversity, Tajima’s D, F_st_, and the observed 1D and 2D site frequency spectra presented in Figure S2, Table S1 were calculated with *dadi*.

Historical changes in population size were inferred with the program *MSMC2*^41^. The analysis was performed 20 times on combined unique sets of 4 strains drawn at random from the Swedish and Zambian populations, respectively. All available SNPs from chromosomes *2R*, *3R*, and *3L* were used for this analysis. The software was used with the following options: msmc2 -p "1*2 + 40*1 + 1*2 + 1*3" -t 6. The “-p” option defines how time is discretized into a number of time segments, for which coalescence rates are estimated. Here we used 47 time-segments but grouped the first 2 and the last 5 in larger segments to compensate for the low signal carried by genetic variation data for very recent and old events. The scaled times and the coalescence rates estimated by *MSMC2* were converted to generations and N_e_, respectively, using a per base-pair mutation rate of 5.21e−9 per bp/per generation. Relative cross-coalescence rates were calculated as 2 * (across population coalescence rate)/(sum of within population coalescence rates).

## Supplementary information

Supplementary Information.

## Data Availability

Short reads have been made available in Genbank (see Supplementary Table 1). All scripts are available at https://gitlab.mpcdf.mpg.de/slaurent/swedish_lines.git.
