# Supplementary material for: Demographic analyses of a new sample of haploid genomes from a Swedish population of Drosophila melanogaster
Source: Sci Rep. 2020 Dec 29;10:22415. doi: 10.1038/s41598-020-79720-1 (PMC7772335; doi:10.1038/s41598-020-79720-1)
Supplement: Supplementary file 1 — Supplementary Information. [file 41598_2020_79720_MOESM1_ESM.pdf]

Demographic analyses of a new sample of haploid genomes from a Swedish population of *Drosophila melanogaster*

Adamandia Kapopoulou[*1], Martin Kapun[*2,3,4], Bjorn Pieper[5], Pavlos Pavlidis[6], Ricardo Wilches[7], Pablo Duchen[8], Wolfgang Stephan[9], Stefan Laurent[§5]

* contributed equally

§ corresponding author: laurent@mpipz.mpg.de

Figure S1

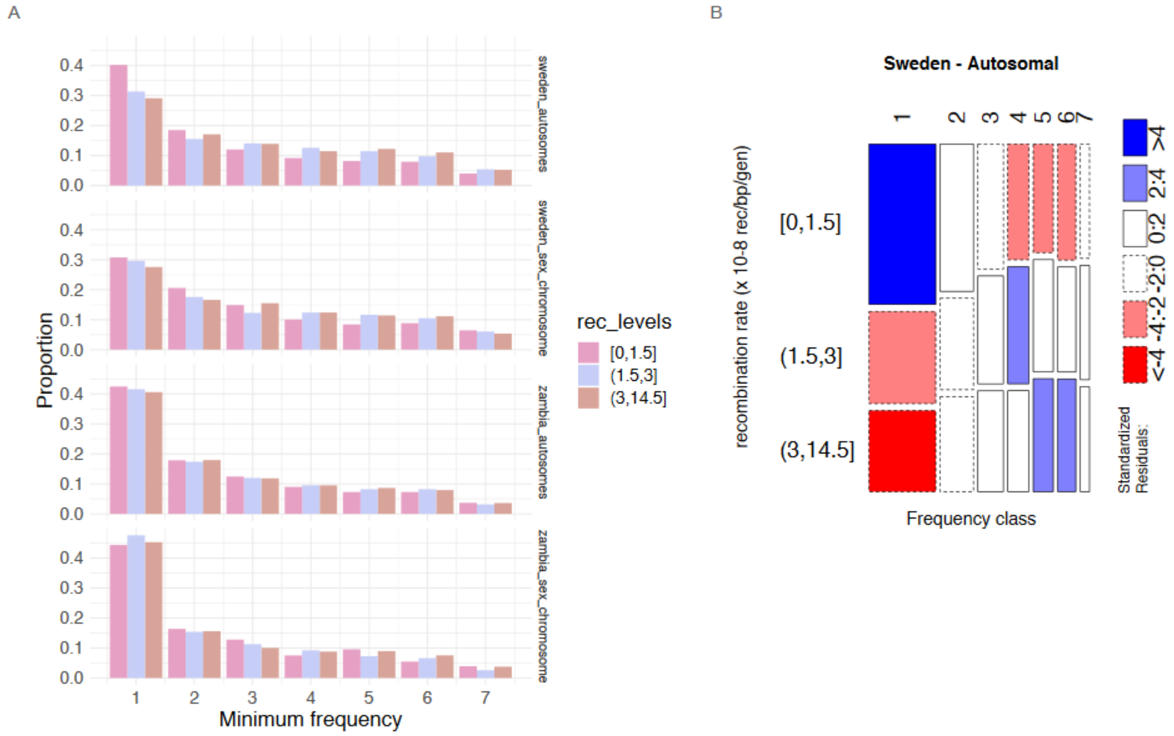

A: Comparison of folded site frequency spectra (SFS) for three genomic recombination classes (low, medium, high). P-values for Pearson's Chi-squared test are indicated and suggest a significant difference between the SFS of different recombination classes for the Swedish autosomal data only. Tests have not been corrected for multiple testing and the P-value for the Swedish X-chromosome data has not been considered significant. **B**: Mosaic-plot displaying the standardized residuals of a loglinear model for the Swedish autosomal data. Frequency classes which are over or under represented compared to the null model (equal SFS for all three recombination classes) are indicated in blue and red, respectively. This analysis indicates an excess in the proportion of singletons in low-recombining chromosomal regions that is consistent with increased linked negative selection. The code source used to generate this figure can be found in the following directory: https://gitlab.mpcdf.mpg.de/slaurent/droso_sweden_pop_genomics.gi.git

Figure S2

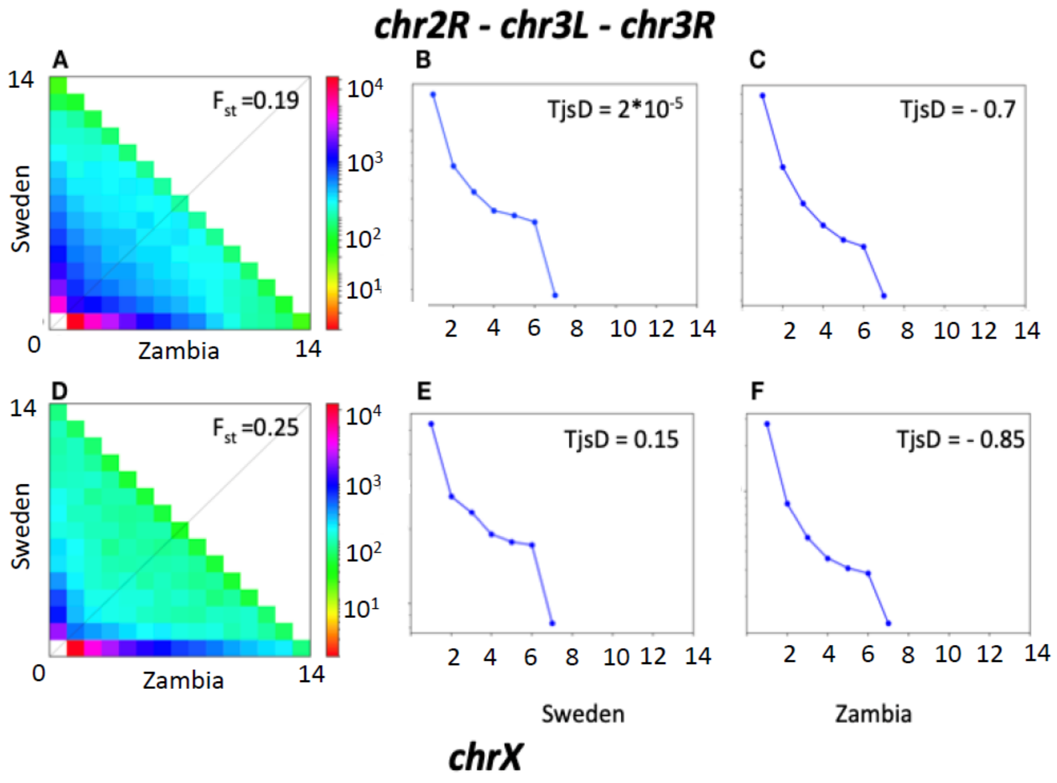

Joint SFS and marginal SFS for the Swedish and Zambian samples: for autosomal (A,B,C) and X-linked data (D,E,F).

Figure S3

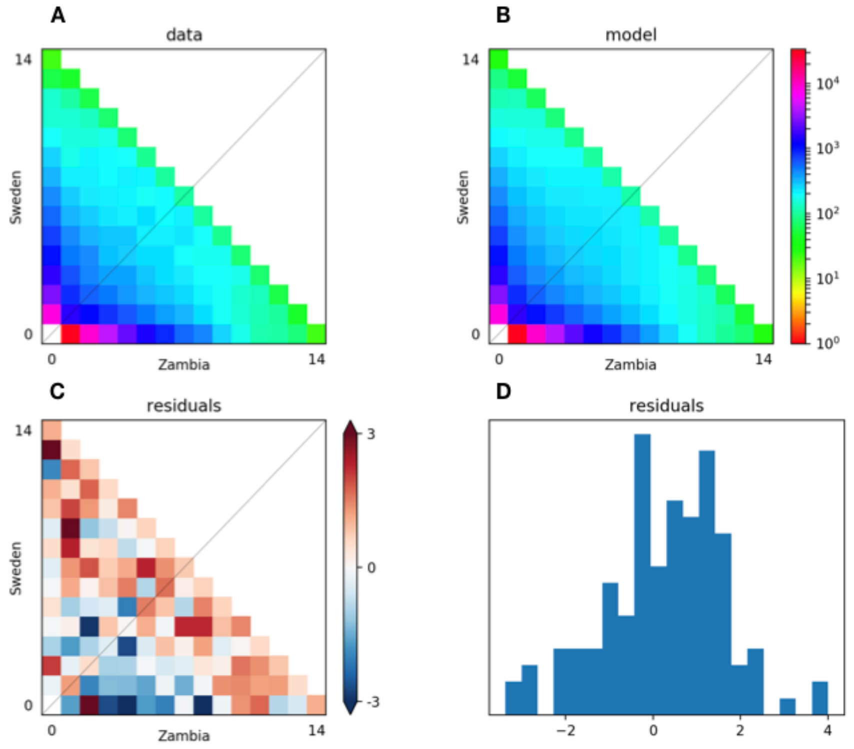

Tajima's D Sweden **data** = 2*10^{-5}
Tajima's D Zambia **data** = - 0.7
F_{st} **data** = 0.19

Tajima's D Sweden **model** = 0.02
Tajima's D Zambia **model** = - 0.6
F_{st} **model** = 0.19

Joint SFS for the observed autosomal dataset (A) and expected under our best model ASYMIG (B) and the residuals obtained for the best model (C, D).

Figure S4

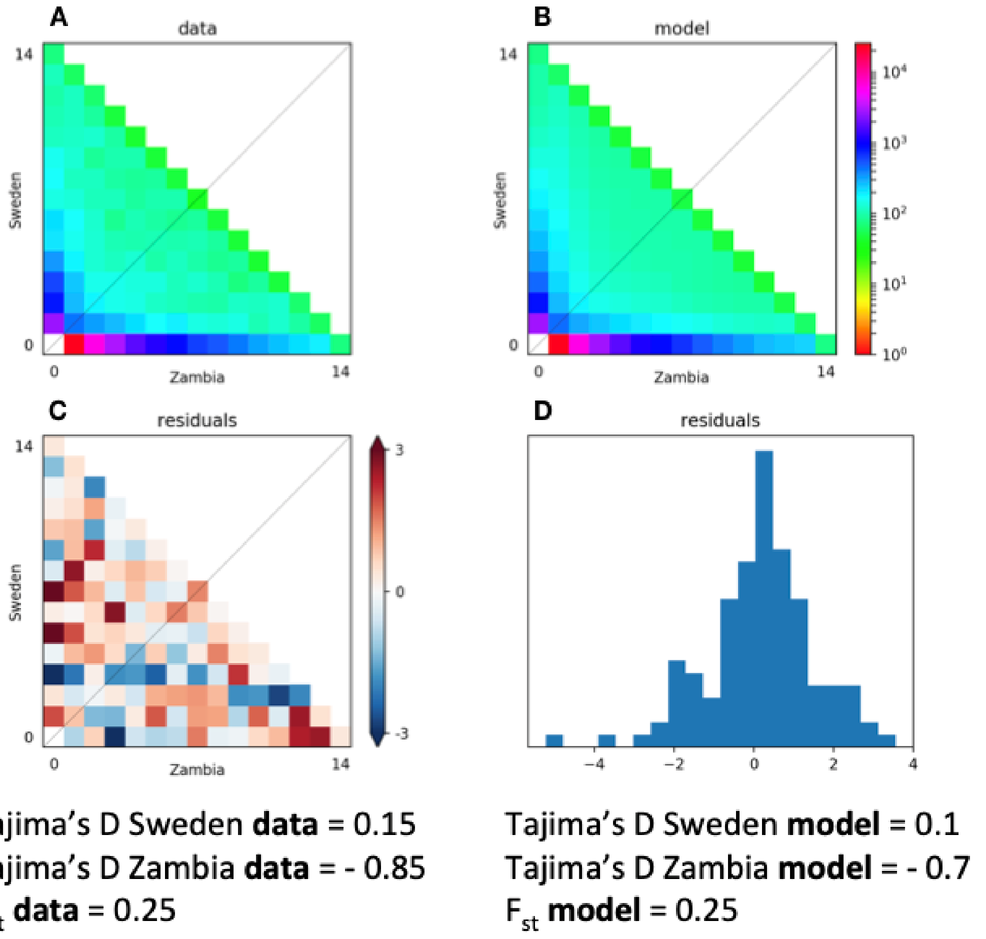

Joint SFS for the observed X-linked dataset (A) and expected under our best model ASYMIG (B) and the residuals obtained for the best model (C, D).

Table S1. **Summaries of genetic diversity**

	Umeå (Sweden)		Siavonga (Zambia)	
	**Autosomes**	**X**	**Autosomes**	**X**
**θ_W (per bp)**	0.003	0.001	0.006	0.003
**θ_π (per bp)**	0.003	0.001	0.005	0.002
**Tajima's D**	$2*10^{-5}$	0.15	-0.7	-0.85
**F_{ST} (Umeå - Siavonga)**	0.19	0.25		

Summary statistics of genetic diversity measured on our neutral dataset (i.e. all introns). All known inversions have been removed as well as chromosome 2L. All statistics have been calculated with *dadi* on the same site frequency spectra used for demographic inference. θ_W : Watterson's estimator of the population mutation rate (θ). θ_π: Tajima's estimator of θ, which corresponds to the average number of differences found in all pairwise comparisons of sequences in the sample (normalised by sequence length here). Tajima's D: The normalised difference between θ_π and θ_W. This statistic is commonly used to quantify how strongly the distribution of allele frequencies (SFS) departs from standard neutral expectations. F_{ST}: the fixation index is a measure of genetic differentiation between populations.

Table S2: Table containing all the parameters inferred by dadi for all four models (NOMIG: no migration, SYMIG: symmetric migration, ASYMIG: asymmetric migration, RASYMIG: asymmetric migration after population divergence

Autosomes

Two tables corresponding to two mutation rates mu and two generation rates

	MCL	AIC	Texp	Tsplit	NEuBot	NEu	NAfr	Nanc	MEtoA	MAtoE	Theta	Tmig
**NOMIG**	-1246	2504	13426	2247	7129	1276071	707153	177615	0	0	14883	NA
**SYMIG**	-678	1370	14175	4384	18306	189559	693697	173695	0,685	0,685	14618	NA
**ASYMIG**	-555	1126	13032	4139	15817	155201	724038	177344	1,23	0,4	14860	NA
**RASYMIG**	-682	1382	3063	3062	56511	61378	733594	181917	1,64	0,65	15310	53395

Total number of sites = 4020733

Nanc = theta/4muL

mu=5.21*10-9

15gen/year

**NOMIG**	-1246	2504	75486	12635	26723	4782969	2650550	665738	0	0	14883	NA
**SYMIG**	-678	1370	79696	24649	68614	710504	2600115	651045	0,685	0,685	14618	NA
**ASYMIG**	-555	1126	73271	23272	59285	581727	2713839	664720	1,23	0,4	14860	NA
**RASYMIG**	-682	1382	17222	17216	211814	230059	2749658	681862	1,64	0,65	15310	53395

mu=1.39*10-9

10gen/year

Table S3: Table containing all the parameters inferred by dadi for all four models (NOMIG: no migration, SYMIG: symmetric migration, ASYMIG: asymmetric migration, RASYMIG: asymmetric migration after population divergence

chrX

Two tables corresponding to two mutation rates mu and two generation rates

	MCL	AIC	Texp	Tsplit	NEuBot	NEu	NAfr	Nanc	MEtoA	MAtoE	Theta	Tmig
**NOMIG**	-1013	2038	5888	795	1112	640088	708483	112823	0	0	10055	NA
**SYMIG**	-511	1037	6335	1453	3048	54177	753981	110441	0,854	0,854	9842	NA
**ASYMIG**	-510	1036	6389	1429	2935	59031	750104	110240	0,78	0,9	9824	NA
**RASYMIG**	-677	1372	2382	2298	26906	19451	777246	112449	1,62	0,78	10021	21685

Total number of sites = 5859268

Nanc = theta/3muL

RASYMIG: Texit + Tmig = Tsplit

mu=5.07*10-9

15gen/year

**NOMIG**	-1013	2038	32216	4349	4056	2334708	2584177	411521	0	0	10055	NA
**SYMIG**	-511	1037	34660	7948	11117	197608	2750133	402830	0,854	0,854	9842	NA
**ASYMIG**	-510	1036	34956	7819	10705	215313	2735989	402098	0,78	0,9	9824	NA
**RASYMIG**	-677	1372	13034	12572	98139	70945	2834991	410155	1,62	0,78	10021	21685

mu=1.39*10-9

10gen/year

Table S4: **Summary statistics of genomic samples.** Table showing information about sampling location and date, short read archive (SRA) accession number, average read depth, false positive rate of SNP calling and karyotype (ST = standard arrangement; INV = inverted arrangement).

**Sample information**					**Coverage**							**Karyotpye**					
**Library**	**Town**	**Country**	**Collection Date**	**SRA Accession**	**X**	**2L**	**2R**	**3L**	**3R**	**AvCov**	**Error rate**	***In(2L)t***	***In(2R)NS***	***In(3L)P***	***In(3R)K***	***In(3R)Mo***	***In(3R)P***
SU02n	Umeå	Sweden	07/2010	SRR2347216	96.5	84.3	86.2	87.1	84.3	87.7	0.0041769	ST	ST	ST	ST	ST	ST
SU05n	Umeå	Sweden	07/2010	SRR2347265	60.9	63.5	57.0	58.2	54.6	58.8	0.0032979	ST	ST	ST	ST	ST	ST
SU07n	Umeå	Sweden	07/2010	SRR2347336	92.8	81.1	81.6	76.6	82.2	82.8	0.0045182	ST	ST	ST	ST	ST	ST
SU08	Umeå	Sweden	07/2010	SRR2347337	49.5	55.2	40.7	46.8	41.3	46.7	0.0029965	INV	ST	ST	ST	ST	ST
SU21n	Umeå	Sweden	07/2010	SRR2347338	78.1	75.8	68.2	72.5	70.8	73.1	0.0045810	INV	ST	ST	ST	ST	ST
SU25n	Umeå	Sweden	07/2010	SRR2347339	67.8	62.6	54.7	62.0	54.9	60.4	0.0051050	ST	ST	ST	ST	ST	ST
SU26n	Umeå	Sweden	07/2010	SRR2347340	63.7	60.1	56.0	59.4	56.5	59.1	0.0030002	ST	ST	ST	ST	ST	ST
SU29	Umeå	Sweden	07/2010	SRR2347341	62.7	50.2	47.2	52.3	47.1	51.9	0.0027015	ST	ST	ST	ST	ST	ST
SU37n	Umeå	Sweden	07/2010	SRR2347342	100.8	82.6	81.8	79.7	73.3	83.6	0.0053593	ST	ST	ST	ST	ST	ST
SU58n	Umeå	Sweden	07/2010	SRR2347343	59.7	52.8	48.9	56.0	47.8	53.0	0.0025252	ST	ST	ST	ST	ST	ST
SU75n	Umeå	Sweden	08/2012	SRR2347308	40.3	30.7	30.8	32.3	31.4	33.1	0.0024483	ST	ST	ST	ST	ST	ST
SU81n	Umeå	Sweden	08/2012	SRR2347331	25.1	22.5	18.9	33.7	34.4	26.9	0.0109843	INV	ST	ST	ST	ST	ST
SU93n	Umeå	Sweden	08/2012	SRR2347333	22.2	20.3	17.6	18.8	18.2	19.4	0.0070109	ST	ST	ST	ST	ST	ST
SU94	Umeå	Sweden	07/2010	SRR2347334	71.7	58.9	55.9	58.5	56.5	60.3	0.0050117	INV	ST	ST	ST	ST	ST
ZI104	Siavonga	Zambia	08/2012	SRR654551	38.0	34.6	31.9	34.5	33.7	34.5	0.0028984	ST	ST	ST	ST	ST	ST
ZI117	Siavonga	Zambia	08/2012	SRR248130	36.0	27.7	25.4	30.3	24.4	28.8	0.0031597	INV	ST	ST	ST	ST	ST
ZI118N	Siavonga	Zambia	08/2012	SRR654664	38.5	31.1	30.2	31.4	30.1	32.2	0.0025102	INV	ST	ST	ST	ST	ST
ZI200	Siavonga	Zambia	08/2012	SRR203234	28.3	28.9	23.9	28.2	25.4	26.9	0.0028224	ST	ST	ST	ST	ST	ST
ZI207	Siavonga	Zambia	08/2012	SRR202075	31.7	35.1	31.2	33.4	32.2	32.7	0.0027042	ST	ST	ST	ST	ST	ST
ZI253	Siavonga	Zambia	07/2010	SRR203350	37.1	39.7	36.3	38.9	38.4	38.1	0.0017713	INV	ST	ST	ST	ST	ST
ZI329	Siavonga	Zambia	07/2010	SRR204006	38.7	40.9	36.7	38.3	38.9	38.7	0.0018525	ST	ST	ST	ST	ST	ST
ZI373	Siavonga	Zambia	08/2012	SRR210782	32.9	32.9	29.5	31.9	31.3	31.7	0.0024303	ST	ST	ST	ST	ST	ST
ZI431	Siavonga	Zambia	08/2012	SRR654556	38.9	39.4	34.3	37.9	37.5	37.6	0.0034443	ST	ST	ST	ST	ST	ST
ZI472	Siavonga	Zambia	07/2010	SRR203465	40.3	40.5	36.2	38.0	38.5	38.7	0.0018797	ST	ST	ST	ST	ST	ST
ZI488	Siavonga	Zambia	08/2012	SRR326792	32.6	30.9	28.5	29.8	29.7	30.3	0.0049452	INV	ST	ST	ST	ST	ST
ZI504	Siavonga	Zambia	08/2012	SRR248124	35.6	28.6	29.1	30.0	27.4	30.1	0.0023286	ST	ST	ST	ST	ST	ST
ZI85	Siavonga	Zambia	08/2012	SRR203508	36.3	39.0	35.4	37.2	37.8	37.2	0.0018799	ST	ST	ST	ST	ST	ST
ZI91	Siavonga	Zambia	08/2012	SRR189423	35.8	35.0	32.3	33.7	33.6	34.1	0.0015601	ST	ST	ST	ST	ST	ST

Supplementary Text: Methods for processing of NGS data

Mapping pipeline: Prior to mapping, we tested raw read libraries in FASTQ format for base quality, residual sequencing adapter sequences and other overrepresented sequences with FASTQC (v0.10.1; http://www.bioinformatics.babraham.ac.uk/projects/fastqc/). We trimmed both the 5' and 3' end of each read for a minimum base quality ≥ 18 and only retained reads with a minimum sequence length ≥ 75 bp using cutadapt version 1.8.3 [1]. We used bbmap (v. 35.50; https://sourceforge.net/projects/bbmap/) with standard parameter values to map intact read pairs, where both reads fulfilled all quality criteria, against a compound reference consisting of the genomes from *D. melanogaster* (v6.12) and genomes from other common pro- and eukaryotic symbionts including *Saccharomyces cerevisiae* (GCF_000146045.2), *Wolbachia pipientis* (NC_002978.6), *Pseudomonas entomophila* (NC_008027.1), *Commensalibacter intestini* (NZ_AGFR00000000.1), *Acetobacter pomorum* (NZ_AEUP00000000.1), *Gluconobacter morbifer* (NZ_AGQV00000000.1), *Providencia burhodogranariea* (NZ_AKKL00000000.1), *Providencia alcalifaciens* (NZ_AKKM01000049.1), *Providencia rettgeri* (NZ_AJSB00000000.1), *Enterococcus faecalis* (NC_004668.1), *Lactobacillus brevis* (NC_008497.1), and *Lactobacillus plantarum* (NC_004567.2) to avoid paralogous mapping of reads belonging to different species. We further filtered for mapped reads with mapping qualities ≥ 20, removed duplicate reads with Picard (v2.17.6; http://picard.sourceforge.net) and re-aligned sequences flanking insertions-deletions (indels) with GATK version 3.4-46 [2]. Distributions of sequencing depths across lines can be found in Figure 1.

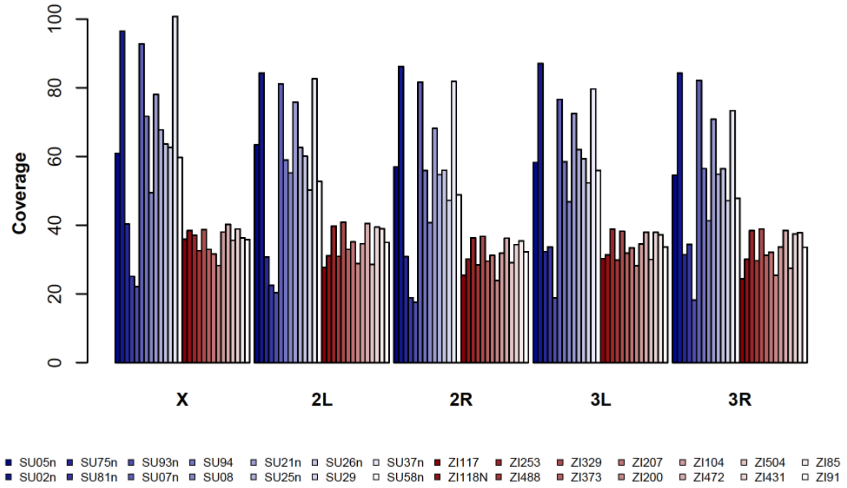

Figure 1: Sequencing depth per line. Bar plots showing the sequencing depths for all 28 samples and 5 chromosomal arms. Line names with the prefix “SU” (blue) and “ZI” (red) indicate the Swedish and Zambian samples, respectively.

Quality control: Since all libraries were constructed from haploid embryos, we assumed that polymorphisms within a library represent either (1) sequencing or (2) mapping errors. Accordingly, we expected to find erroneous alleles only at very low frequencies in each dataset. Alternatively, any problem during the construction of haploid embryos would lead to diploid sequences that result in residual heterozygosity characterized by an excess of polymorphisms with frequencies close to 0.5 in the affected library. To test for these hypotheses, we investigated the distribution of minor – putatively erroneous – allele frequencies for each library separately. In addition, we divided the number of erroneous alleles by the total coverage at variant and invariant positions to calculate library-specific error-rates. Distribution of sample specific error-rates can be found in Figure 2.

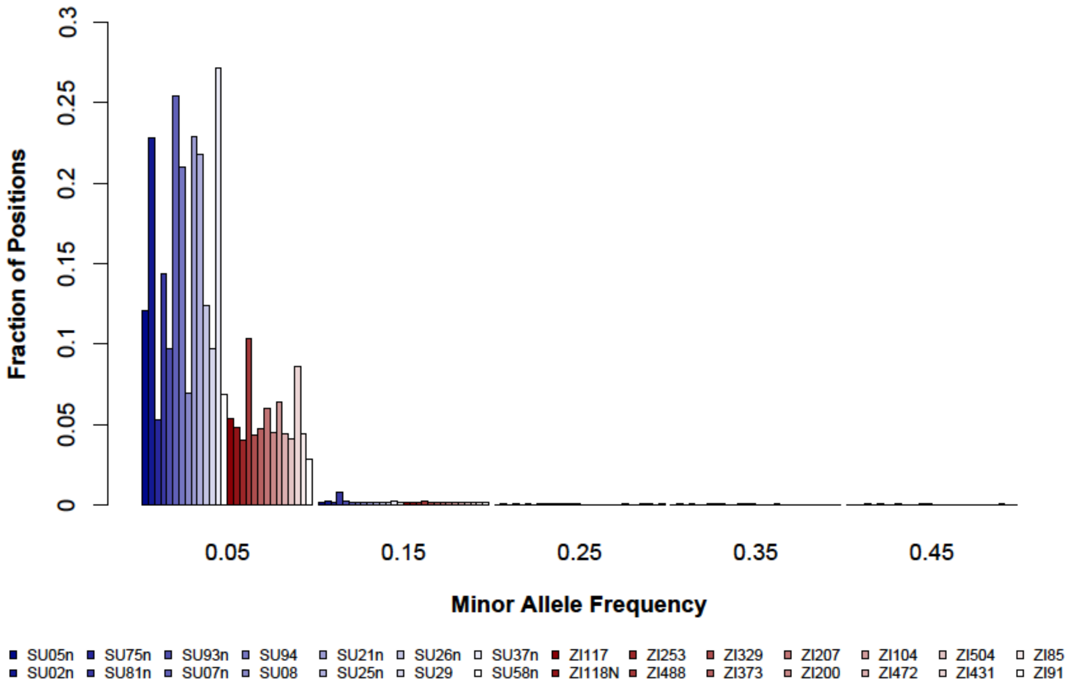

Figure 2: Sample-specific error rates. Barplots showing the sample-specific frequencies of false positive alleles due to sequencing or mapping errors. The y-axis shows the proportion of total positions that contain false position alleles of the corresponding frequency class.

Variant calling: We identified single nucleotide polymorphisms (SNPs) based on a combination of stringent heuristic criteria to exclude sequencing and mapping errors in each of the Swedish and Zambian datasets using custom software: For each library, we excluded polymorphic positions with minor frequencies > 0.1. In all other cases, we considered the major allele as the correct allelic state for a given individual. To avoid erroneous SNPs due to inflated sampling error at low-coverage sites or due to paralogous alleles at sites with excessive coverage from mapping errors, we only considered positions with more than 10-fold and less than 200-fold coverage. We further ignored positions where less than 14 of the 28 samples (14 Swedish and 14 Zambian) fulfilled the above-mentioned quality criteria. At last, we refined the SNP dataset by excluding SNPs located either within known transposable elements (TE) based on the *D. melanogaster* reference genome (v.6.12) or within a 5-base pair distance to indel polymorphisms supported by 10 reads across all samples. Finally, the same set of filters was applied to the full dataset (polymorphic and monomorphic positions) to generate mask files for missing data for *MSMC2* and the total number of monomorphic sites in our dataset, which is required for demographic inference with *dadi* [3, 4]. Missing data proportion per line can be found in Figure 3.

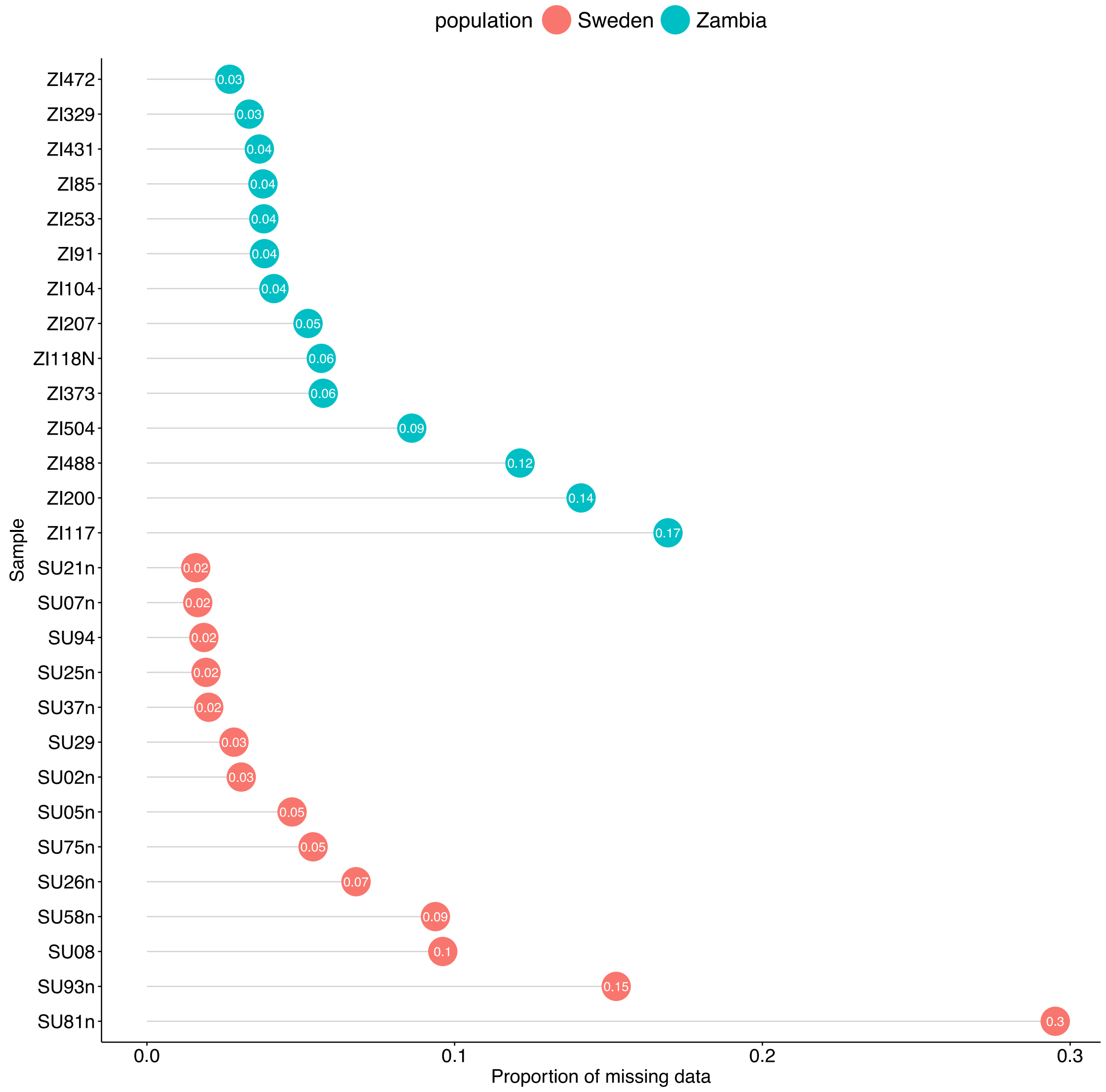

Figure 3: Proportion of missing data for each sample.

Bioinformatic karyotyping: Following the approach in Kapun et al.[5], we used a panel of karyotype-specific marker SNPs that are diagnostic for seven chromosomal inversions (*In(2L)t, In(2R)NS, In(3L)P, In(3R)C, In(3R)K, In(3R)Mo* and *In(3R)Payne*) to karyotype all Swedish samples based on presence or absence of alleles which are in tight linkage with the corresponding inversion (Table S3). We further used the same method to confirm the inversion status in previously karyotyped samples from Zambia. We only considered a sample to be positive for an inversion if it carried ≥ 95% of all alleles that are specific to the corresponding inversion.

References:

1. Martin, M. Cutadapt removes adapter sequences from high-throughput sequencing reads. *EMBnet.journal* **17**, 10–12 (2011).
2. McKenna, A. *et al.* The Genome Analysis Toolkit: A MapReduce framework for analyzing next-generation DNA sequencing data. *Genome Res.* **20**, 1297–1303 (2010).
3. Laurent, S. *et al.* The population genomics of rapid adaptation: disentangling signatures of selection and demography in white sands lizards. *Mol. Ecol.* **25**, 306–323 (2016).
4. Gutenkunst, R. N., Hernandez, R. D., Williamson, S. H. & Bustamante, C. D. Inferring the Joint Demographic History of Multiple Populations from Multidimensional SNP Frequency Data. *PLoS Genet.* **5**, e1000695 (2009).
5. Kapun, M., van Schalkwyk, H., Bryant, M., Flatt, T. & Schlötterer, C. Inference of chromosomal inversion dynamics from Pool-Seq data in natural and laboratory populations of Drosophila melanogaster. *Mol. Ecol.* **23**, 1813–1827 (2014).
